# Emerging therapies in Ewing sarcoma

**DOI:** 10.1097/CCO.0000000000001048

**Published:** 2024-05-22

**Authors:** Sandra J. Strauss, Pablo Berlanga, Martin G. McCabe

**Affiliations:** a Department of Oncology, University College London Cancer Institute, London, United Kingdom; b Department of Pediatric and Adolescent Oncology, Gustave-Roussy, Université Paris-Saclay, Villejuif, France; c Division of Cancer Sciences, Faculty of Biology, Medicine and Health, University of Manchester, Manchester, UK

**Keywords:** collaborative trials, Ewing sarcoma, tyrosine kinase inhibitors

## Abstract

**Purpose of review:**

There is an unmet need to improve outcomes for patients for Ewing sarcoma, a rare, aggressive sarcoma with a peak incidence in adolescents and young adults (AYA). Current therapy at diagnosis involves multiagent chemotherapy and local therapy, but despite intensification of treatment, those with metastases at diagnosis and recurrent disease have poor outcomes.

**Recent findings:**

Improved understanding of Ewing sarcoma biology has identified novel targets with promising activity in Ewing sarcoma patients, including tyrosine kinase inhibitors that are now undergoing evaluation as combination and maintenance therapy. Other emerging therapies include those that target the EWSR1::FLI1 fusion oncoprotein, and act on DNA damage, cell cycle and apoptotic pathways. Immunotherapeutic approaches, particularly CAR-T-cell therapy directed at GD2, also hold promise. Recent collaborative clinical trials that have defined an international standard of care for patients with newly diagnosed Ewing sarcoma and novel platform studies with adaptive designs offer unique opportunities to investigate these therapies inclusive of all ages.

**Summary:**

Close international collaboration between clinicians and biologists will allow us to prioritize promising emerging therapies and develop biomarkers to facilitate their incorporation into standard of care and more rapidly translate into benefit for Ewing sarcoma patients.

## INTRODUCTION

Ewing sarcoma is an aggressive sarcoma that can arise in bone or soft tissue and has a peak incidence in adolescents and young adults (AYA). Treatment of Ewing sarcoma requires multimodality therapy that involves intensive multiagent chemotherapy and local therapy (surgery, radiation therapy, or both). With an incidence of 1.5 per million in the AYA population, treatment advances in this rare disease have largely resulted from national and international collaborative clinical trials, which have aimed to address questions around chemotherapy and better define risk groups, to allow more tailored treatment strategies. Within this setting, event-free survival for patients with localized disease now approaches 75%; however, therapy is associated with substantial acute and long-term toxicities and patients with metastatic or recurrent disease continue to have poor outcomes. Therefore, there remains a significant unmet need to better tailor treatment and identify novel therapies to improve outcome.

Ewing sarcoma is defined by a pathognomonic translocation of the *EWSR1* gene with one of the ETS family of genes, most commonly EWSR1::FLI1 [1]. To date, attempts at direct targeting of the fusion onco-protein have not successfully translated into the clinic but its identification has advanced our understanding of Ewing sarcoma oncogenesis and biology and provided a focus for development of targeted therapeutics [2]. This, coupled with improved collaboration between Ewing sarcoma paediatric and medical oncologists and researchers has led to better defined standard of care and development of platforms to provide a backbone upon which to layer and evaluate novel agents. These trials, as well as studies focused on emerging therapies that include multitargeted tyrosine kinase inhibitors, therapies that target the oncoprotein, DNA damage and cell cycle pathways and immunotherapy strategies will be discussed. 

**Box 1:**
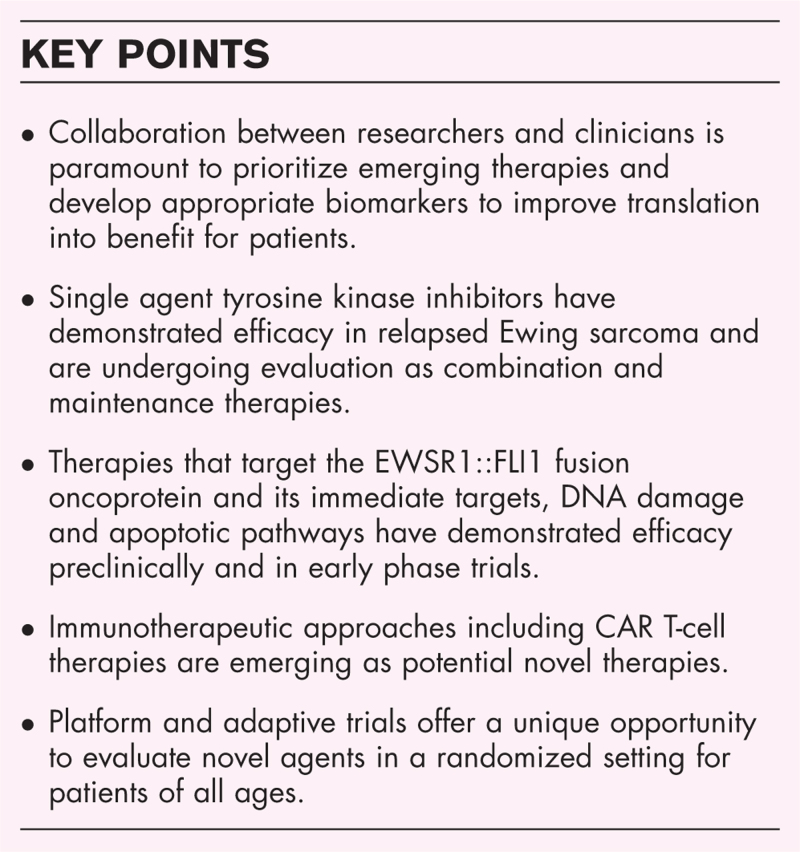
no caption available

## CURRENT STANDARD OF CARE

### First-line therapy

Until recently, an international standard chemotherapy for Ewing sarcoma had not been defined with different regimens employed in Europe and the United States for patients with newly diagnosed disease. In this setting, the EuroEwing Consortium (EEC) trial, Euro-Ewing 2012, compared therapy based on the previous EURO-EWING 99 trial that used induction chemotherapy [vincristine, ifosfamide, doxorubicin, and etoposide (VIDE) prior to local control] followed by risk-adapted randomized treatment, with the Children's Oncology Group (COG) protocol, interval compressed vincristine, doxorubicin, and cyclophosphamide (VDC), ifosfamide and etoposide given bi-weekly, which had demonstrated favourable outcomes for patients with localised disease [3,4]. The study, which randomized 640 patients, demonstrated interval compressed VDC/IE to be more effective and less toxic than its comparator for all stages of newly diagnosed disease and defined it to be the standard of first-line care for all patients with Ewing sarcoma [5^▪▪^]. Recent studies from the COG, failed to demonstrate improved outcomes with the addition of cyclophosphamide and topotecan to interval compressed VDC/IE in patients with localized disease, or the IGFR inhibitor, ganitumab, in patients with metastatic disease [6,7]; however, these studies demonstrated the feasibility of the use of this regimen as a backbone and platform upon which to evaluate novel agents in the first-line setting. The current European collaborative trials, Inter-Ewing-1 and iEuroEwing are evaluating the addition of cyclophosphamide and vinorelbine to VDC/IE as maintenance chemotherapy in this setting but with flexible designs that allow evaluation of additional agents as described forthwith.

### Recurrent disease

Patients with recurrent or refractory disease have poor outcomes with a median overall survival of around 12 months [8]. Multiple regimens are used in this setting, albeit with historically poor-quality evidence. The rEECur phase II/III adaptive multiarm trial is the first to compare regimens in a randomized design. To date, four have been evaluated: topotecan with cyclophosphamide (TC), irinotecan with temozolomide (IT), gemcitabine with docetaxel (GD) and high-dose infusional ifosfamide (IFOS). The trial is ongoing. In pairwise comparisons, GD was the least effective; imaging response and survival outcomes after TC were marginally better than after IT, whereas in a phase III comparison, IFOS had significantly better progression-free survival (PFS) and overall survival (OS) than TC, making IFOS the most effective regimen, albeit with significant renal and neurological toxicity in a minority (Table 1) [9,10^▪▪^]. With over 570 patients recruited to date, rEECur is a paradigm of effective and efficient trial design in a rare cancer setting and is currently comparing IFOS, carboplatin with etoposide and IFOS with the addition of an emerging therapy, the tyrosine kinase inhibitor (TKI) lenvatinib as described in the following section.

**Table 1:** rEECur: pairwise comparisons of chemotherapy combinations in recurrent and refractory Ewing sarcoma^a^

Chemotherapy comparison	Number in comparison	Number receiving each regimen	Hazard ratio for EFS (95% CrI)	Confidence	Comparison favours	Median EFS^b^
IT/TC	230	115/115	1.12 (0.84–1.48)	78%	TC	
IFOS/TC	146	73/73	0.69 (0.48–0.99)	98%	IFOS	IFOS 5.7 months (95% CI 3.8–6.9 months) vs. TC 3.5 months (95% CI 2.1–5.1 months)
GD/TC	125	65/60	1.60 (1.08 – 2.36)	>99%	TC	
GD/IT	117	58/59	1.40 (0.93–2.10)	95%	IT	
IFOS/IT	80	38/42	1.00 (0.61–1.63)	50%	–	
IFOS/GD	47	22/25	0.55 (0.28–1.07)	96%	IFOS	

CrI, credible interval; EFS, event-free survival; GD, gemcitabine with docetaxel; IFOS, high-dose ifosfamide; IT, irinotecan with temozolomide; TC, topotecan with cyclophosphamide.

a Data presented at the Connective Tissue Oncology Society annual meeting 2022.

b Data presented at American Society of Clinical Oncology annual meeting 2022.

## EMERGING THERAPIES

### Tyrosine kinase inhibitors

Aberrant angiogenesis is crucial for Ewing sarcoma growth and metastasis and preclinical data supports this target in Ewing sarcoma [11]. TKIs with antiangiogenic activities such as regorafenib and cabozantinib have shown encouraging single-agent results in single-arm and randomized trials versus placebo with 10–26% objective tumour responses and median PFS of 3–4.5 months in heavily pretreated patients, mostly adults [12^▪▪^,^13^^▪▪^,^14^]. In these studies, TKI single-agent antitumour activity appears similar to conventional salvage chemotherapy, although never been tested in a randomized trial (Table 2). TKI activity in children and adolescents is much less explored, with no antitumour activity of cabozatinib in a limited cohort of patients with Ewing sarcoma treated within a COG phase 2 trial [15]. Combinations of TKI with several agents are currently ongoing including with IFOS, checkpoint inhibitors at disease recurrence and as maintenance therapy [16,17^▪^,18^▪^,^19^] (Table 3).

**Table 2:** Single agent tyrosine kinase inhibitor trials in recurrent, relapsed, Ewing sarcoma

Tyrosine kinase inhibitor	Trial name	Age inclusion (years)	No. patients^a^	Media age, years (range)	RECIST 1.1 ORR, % (95% CI)	Median PFS, months (95% CI)
Cabozantinib	CABONE	>12	39	36 (23–45)	26 (13–42)	4.4 (3.7–5.6)
Regorafenib	REGOBONE	≥18	23	32 (18–59)	13 (NR) [placebo, 7 (NR)]	2.6 (1.1–5.3) [placebo 0.9 (0.8–1.7)]
Regorafenib	SARC024	≥18	30	32 (19–65)	10 (NR)	3.45 (1.7–3.7)

CI, confidence interval; NR, not reported; ORR, overall response rate; PFS, progression-free survival.

a Assessable.

**Table 3:** Ongoing trials of emerging therapies in Ewing sarcoma

Target/agent	Trial number	Title	Ref
EWS:: ETS			
Fusion oncoprotein	NCT04067115	SARC037: A Phase I/II Study to Evaluate the Safety of Trabectedin in Combination With Irinotecan in Ewing Sarcoma Patients	[27]
CDK 9-mediated transcriptional inhibition	NCT03604783	First-in-Human Study of Oral TP-1287 in Patients with Advanced Solid Tumors including Ewing sarcoma	[30]
Tyrosine kinase inhibitors (TKIs)			
Lenvatinib	ISRCTN36453794	rEECur – International Randomised Controlled Trial of Chemotherapy for the Treatment of Recurrent and Primary Refractory Ewing Sarcoma	[10^▪▪^]
Regorafenib	NCT05830084	Phase Ib Regorafenib With Conventional Chemotherapy/Newly Diagnosed Patients/Multimetastatic Ewing Sarcoma	[20^▪▪^]
Regorafenib	NCT04698785	Efficacy of Regorafenib Combined With Best Supportive Care as Maintenance Treatment in High Grade Bone Sarcomas Patients	[17^▪^]
Regorafenib	NCT04055220	Efficacy and Safety of Regorafenib as Maintenance Therapy After First-line Treatment in Patients With Bone Sarcomas.	[18^▪^]
Cabozantinib	NCT05182164	Combination of Pembrolizumab and Cabozantinib in Patients With Advanced Sarcomas (PEMBROCABOSARC)	[16]
Cabozantinib	NCT06156410	Cabozantinib With Ifosfamide in Ewing's Sarcoma and Osteosarcoma	[19]
DNA damage, cell cycle and apoptosis			
PARP +ATR inhibitor	NCT02813135	European Proof-of-Concept Therapeutic Stratification Trial of Molecular Anomalies in Relapsed or Refractory Tumors (ESMART): Arm N: Olaparib and Ceralasertib	[41^▪^]
CDK4/6	NCT03709680	Study of Palbociclib Combined With Chemotherapy in Pediatric Patients With Recurrent/Refractory Solid Tumors	[45]
CDK4/6	NCT05440786	CAMPFIRE: A Study of Abemaciclib (LY2835219) in Participants With Ewing's Sarcoma	[44]
DR5 agonist	NCT03715933	The Tetravalent Death Receptor 5 Agonist Inhibrox-109 combined with chemotherapy in Ewing sarcoma	[47^▪▪^]
CAR T-cell therapy trials			
GD2 CART	NCT03635632	C7R-GD2.CART Cells for Patients With Relapsed or Refractory neuroblastoma and Other GD2 Positive Cancers (GAIL-N)	[51^▪^]
EGFR CAR T	NCT03618381	EGFR806 CAR T Cell Immunotherapy for Recurrent/Refractory Solid Tumors in Children and Young Adults	[52^▪^]
GD2 CAR T	NCT03373097	Anti-GD2 CAR T Cells in Pediatric Patients Affected by High Risk and/or Relapsed/Refractory Neuroblastoma or Other GD2-positive Solid Tumors	[53^▪^]

Currently the EEC is testing the combination of lenvatinib with IFOS within the rEECur trial as described above and regorafenib in newly diagnosed patients. The main concerns of the potential combination of regorafenib with current first-line Ewing sarcoma standard treatment are overlapping toxicities such as cardiac toxicity, increased haematological toxicity that may impair chemotherapy dose intensity or combination with radiotherapy. Therefore, this combination is currently being explored in a phase Ib trial in patients with newly diagnosed metastatic Ewing sarcoma (other than lung/pleura) for the first time bringing a TKI into first-line therapy and, based on its results, is planned to be latter explored in the randomized INTER-EWING-1 trial (VDC/IE ± regorafenib) [20^▪▪^,^21^^▪▪^].

### Targeting EWSR1::FLi1

Several strategies have attempted to directly or indirectly target the *EWSR1::FLI1* gene fusion or immediate downstream targets. YK-4-279 and its clinical derivative TK-216 were developed in Ewing sarcoma based on their supposed ability to inhibit the interaction between EWSR1::FLI1 and an effector molecule RNA helicase A. In preclinical models, YK-4-279 functionally inhibited EWSR1::FLI1 activity, Ewing sarcoma cell and xenograft growth and caused G2/M arrest and apoptosis [22]. In combination studies, vinca alkaloids were synergistic, reducing tumour burden and prolonging survival in Ewing sarcoma xenografts [23]. However, clinical results were disappointing, a phase I/II study of TK216 alone or with vincristine demonstrating responses in less than 10% of patients, and subsequent analyses suggested TK216's primary activity was as a conventional cytotoxic via destabilization of microtubules rather than inhibition of EWSR1::FLI1 [24,25]. Despite its great initial promise, the agent is no longer being developed in Ewing sarcoma.

The DNA minor groove-binding alkylating cytotoxic agent trabectedin is highly potent *in vitro* against Ewing sarcoma cells and other fusion protein-driven sarcomas, with direct modulatory effects on transcriptional targets of EWSR1::FLI1, yet has little to no single agent clinical activity (Baruchel [26]). However, by hijacking the transcriptional effects of EWSR1::FLI1 on the Werner syndrome helicase *WRN*, Ewing sarcoma cells can be made hypersensitive to the camptothecin irinotecan, leading to cooperative activity between irinotecan and trabectedin preclinically, and an encouraging 29% response rate in the phase I SARC037 trial in Ewing sarcoma patients treated at the recommended combination phase 2 dose [27,28^▪▪^]. A combination phase 2 expansion of trabectedin and irinotecan and a single agent study of the more potent sister compound lurbinectedin are ongoing.

Interest has grown in the cyclin-dependent kinases that facilitate gene transcription: CDK7, 9, 12 and 13, involved in transcriptional pause release, elongation and termination. Ewing sarcoma are potentially dually vulnerable to transcriptional CDK inhibition: ubiquitous binding of EWSR1::FLI1 at promoter sites results in widespread enhancer re-programming and transcriptional addiction, and the EWSR1::FLI1 transcript itself is relatively long, at more than 4 kb in length [29]. A phase I trial of the CDK9 inhibitor is recruiting Ewing sarcoma patients; results are awaited [30]. Preclinical data suggests a synthetic lethality relationship between EWSR1::FLI1 expression and CDK12/13 inhibition, with additional synergy resulting from co-treatment with PARP inhibitors [31]. Similarly, co-inhibition of epigenetic and transcriptional machinery with H3K27 demethylase and CDK7 inhibitors is reported to result in synergistic inhibition of Ewing sarcoma preclinical models [32].

The widespread epigenetic re-programming of Ewing sarcoma cells by EWSR1::FLI1 results in substantial heterogeneity and consistent enhancer hypomethylation. Inhibitors of lysine-specific demethylase 1 (*LSD1*) represent an opportunity to control Ewing sarcoma tumour growth through widespread alterations in gene expression and have shown promise in preclinical studies [33]. Unfortunately, an early Ewing sarcoma-specific trial of the inhibitor INSC059872 terminated early and development of a second, seclidemstat, has been halted.

### DNA damage, cell cycle and apoptotic pathways

#### Poly(ADP-ribose) polymerase 1 inhibition

The DNA damage response protein, poly(ADP-ribose) polymerase 1 (PARP1), was identified as a potential target in Ewing sarcoma following demonstration that PARP1 is a transcriptional coregulator of EWS-FLI1 [34] and that the translocation is a genetic determinant of PARP sensitivity in cellular models. A phase II study of single agent, Olaparib failed to demonstrate clinical activity [35]; however, strong evidence that PARP inhibitors were also able to selectively potentiate the activity of DNA-damaging cytotoxic agents such as irinotecan and temozolomide in Ewing sarcoma animal models led to several phase I and II combination clinical trials [36–38]. All trials demonstrated tantalizing hints of clinical activity in a heavily pretreated, relapsed patient population; however, toxicity, mainly myelosuppression, limited the opportunity to dose intensify these combination therapies and failed to demonstrate an obvious advantage over higher dose, standard combination cytotoxic therapy. One recent trial, of Olaparib in combination with irinotecan within the E-MSART platform demonstrated overall response rate ORR of 2/26 (8%) and 7 with SD (27%), within the platform; however, molecular enrichment did not predict response; and identification of predictive biomarkers for PARPi cytotoxic combination trials remains warranted for ongoing development [39]. Noncytotoxic agents may be better potential partners for PARP inhibition. DNA repair is dependent on DNA damage response pathways, particularly through ataxia telangiectasia and Rad3-related protein (ATR), which is activated in response to replication stress, a notable feature of Ewing sarcoma through EWS-FLI1-mediated increased transcription [40]. An ATR-PARP inhibitor combination strategy is, therefore, attractive, with this combination recently observed to be tolerable in an ongoing paediatric phase I trial performed within the E-MSART platform [41^▪^]. The results of the phase II expansion that includes Ewing sarcoma patients are awaited with interest.

#### Cell cycle and apoptotic pathway targets

A functional, chemical genomic and super-enhancer screen identified CDK4 as a potential vulnerability in Ewing sarcoma, with pharmacologic inhibition using CDK4/6 inhibitors leading to cell death in Ewing sarcoma preclinical models, supporting exploration of CDK4/6 inhibitors as a therapeutic approach for Ewing sarcoma patients [42,43]. This has led to ongoing trials evaluating IT ± palbociclib in children and adolescents with Ewing sarcoma and IT ± abemaciclib in Ewing sarcoma patients up 39 years [44,45].

Binding of TNF-related apoptosis-inducing ligand (TRAIL) to death receptors DR4 and/or DR5 leads to TRAIL-mediated apoptosis in many cancers including sarcomas, with Ewing sarcoma cells exhibiting particular sensitivity to this pathway. Development of clinically tolerable DR agonists, such as the tetravalent DR5 agonist antibody, INBRX-109, has resurrected interest in this target in sarcomas [46]. Upregulation of DR by chemotherapy that enhances TRAIL effectiveness provides the rationale for a phase I trial of this agent, in combination with Irinotecan Temozolomide in round cell sarcomas. Preliminary results are encouraging with disease control observed in five of seven of Ewing sarcoma patients, and clinical benefit over 6 months in 30.8% of patients [47^▪▪^].

### Immunotherapy and CAR T-cell therapy

Immunotherapies aim at inducing antitumor immune response by a nontumor-specific immune activity (e.g. immune checkpoint inhibitors) or by targeting tumour-specific features (e.g.: monoclonal antibody, CAR T cells and cancer vaccines). Although they have been associated with very positive results in many adult cancers, their benefit in sarcomas and paediatric/AYA cancers is still limited. In Ewing sarcoma, immune checkpoint inhibitors, did not result in significant clinical activity [48–50]. Gangliosides and B7-H3 are surface antigens that are expressed by many paediatric solid tumours, such as neuroblastoma and some sarcomas including Ewing sarcoma. The benefit of the use of anti-GD2 as well as of anti-B7-H3 monoclonal antibodies have been studied as single agents in early phase clinical trials, but although the trials are completed, results have not been shared.

T cells engineered to express a CAR (CAR T cells) can be also used to effectively target tumour cells and hold promise in many cancers. The administration of GD2 or EGFR CAR T cell is under investigation in solid tumours, with four early clinical trials underway in Ewing sarcoma [51^▪^–54^▪^]. One, a trial of a GD2-CAR, recently demonstrated exciting activity in patients with relapsed neuroblastoma; the results in Ewing sarcoma are eagerly awaited [55^▪^]. Other studies use CARs to enhance the activity of natural killer (NK) cells against Ewing sarcoma. A phase I/II study consisting of infusion of allogeneic haploidentical NK cells in combination with chemotherapy and/or radiotherapy and a phase I trial of memory T cells expressing an NKG2D are currently recruiting as a treatment for paediatric, adolescent and young adult patients with refractory sarcoma. In the next few years, more preclinical knowledge on the benefit of TCR-transduced T cells as well as of bispecific T-cell engagers will be available to design future clinical studies in Ewing sarcoma [56,57]. Immunization of patients using peptides, full-length proteins or tumour cell lysates against Ewing sarcoma-associated antigens has been explored, with few encouraging results [58]. The Vigil immunotherapy, consisting of autologous tumour cells transfected with a plasmid expressing recombinant human granulocyte macrophage-colony stimulating factor and bifunctional short hairpin RNA against furin to elicit tumour-specific systemic immune response, has been tested in a phase I study in patients with Ewing sarcoma. The treatment was well tolerated and was associated with an objective response in two of eight patients [59]. However, the phase III randomized study of intradermal autologous Vigil immunotherapy in combination with irinotecan and temozolomide versus chemotherapy therapy alone in patients with metastatic, refractory or progressive Ewing sarcoma was recently closed due to slow accrual [57].

The overall disappointing results of these approaches in Ewing sarcoma are at least partially because of the low mutational burden and a resulting lack of high-affinity neoepitopes, the lack of potentially tumour-reactive T cells in the tumour tissue and HLA loss, as well as accumulation of immunosuppressive cells, mainly macrophages, in the tumour microenvironment [60]. The successful application of future immunotherapies in Ewing sarcoma will likely depend on combinatorial strategies that would improve the antitumor immunity while reverting the immunosuppressive tumour microenvironment.

## CONCLUSION AND FUTURE DIRECTIONS

Development of novel targets remains a challenge in Ewing sarcoma, but improved understanding of biology and the immune microenvironment are leading to more targeted approaches with promising preliminary data in early phase clinical trials. An international standard of care for newly diagnosed Ewing sarcoma patients, and novel trial platforms provide unique opportunities to evaluate these emerging therapies in larger cohorts of patients inclusive of age. Increased collaboration between clinicians and researchers to prioritize these, as well as a focus on risk stratification and identification of biomarkers is required to ensure rapid translation into standard-of-care therapies for patients.

## Acknowledgements

*None.*

### Financial support and sponsorship

*S.J.S. was funded in part by the NIHR UCLH Biomedical Research Centre.*

### Conflicts of interest

*S.J.S. has received funding from Ceridwen Oncology, Boehringer Ingelheim, Iinhibrx and Tessellate Bio. P.B. has received institutional funding from EUSA Pharma/Recordati and institutional funding and drugs for trials from Bayer. M.G.M. has received institutional funding from Eisai, Amgen and Ipsen.*

## REFERENCES AND RECOMMENDED READING

Papers of particular interest, published within the annual period of review, have been highlighted as:

▪ of special interest
▪▪ of outstanding interest
